# Supplementary material for: Growth phase-dependent reorganization of cryptophyte photosystem I antennae
Source: Commun Biol. 2024 May 11;7:560. doi: 10.1038/s42003-024-06268-5 (PMC11088674; doi:10.1038/s42003-024-06268-5)
Supplement: Supplementary file 2 — Supplementary information [file 42003_2024_6268_MOESM2_ESM.pdf]

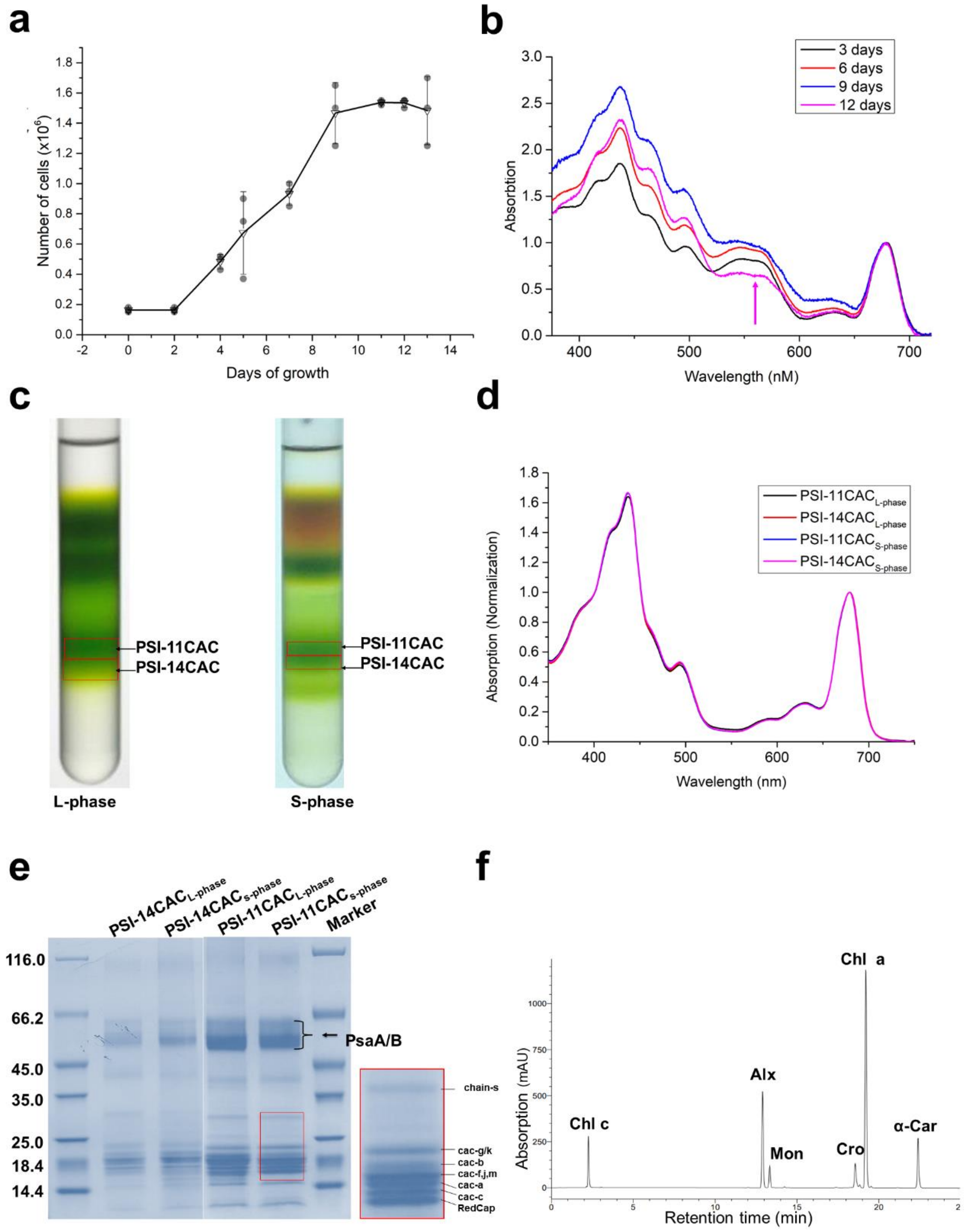

Supplementary Figure 1. Purification and characterization of PSI-CAC complexes. **a.** The growth curve of *R. salina* cells. Cells enter the stationary growth phase at the ninth day. Cell numbers (indicated by triangles) were averaged from three biological replicates. **b.** The absorption spectra of *R. salina* cells (cultured for 12 days) measured every three days. The decrease of PE545 content in cells in the late stationary growth phase (12 days) is indicated by the reduced absorption at 545 nm (indicated by a

magenta arrow). **c.** Isolation of PSI-CAC complexes from cells in both logarithmic and stationary growth phase by sucrose density gradient centrifugation. The green bands corresponding to PSI-CAC samples were divided into the upper half (labeled as PSI-11CAC) and the lower half (labeled as PSI-14CAC), and collected separately. **d.** Absorption spectra of four types of PSI-CAC complexes. **e.** SDS-PAGE analysis of PSI-CAC complexes. The protein composition of Coomassie bands was identified based on the mass spectrometry analysis. **f.** Analysis of the pigment composition of PSI–14CAC$_{L\text{-}phase}$ complex by HPLC, recorded at 440 nm. Six major pigment peaks were identified as chlorophyll c (Chl c), Alloxanthin (Alx), Monadoxanthin (Mon), Crocoxanthin (Cro), Chlorophyll a (Chl a) and α-Carotene (α-Car), based on the characteristic absorption spectrum of each peak fraction. The representative results were shown from at least two biological replicates in **b-f**.

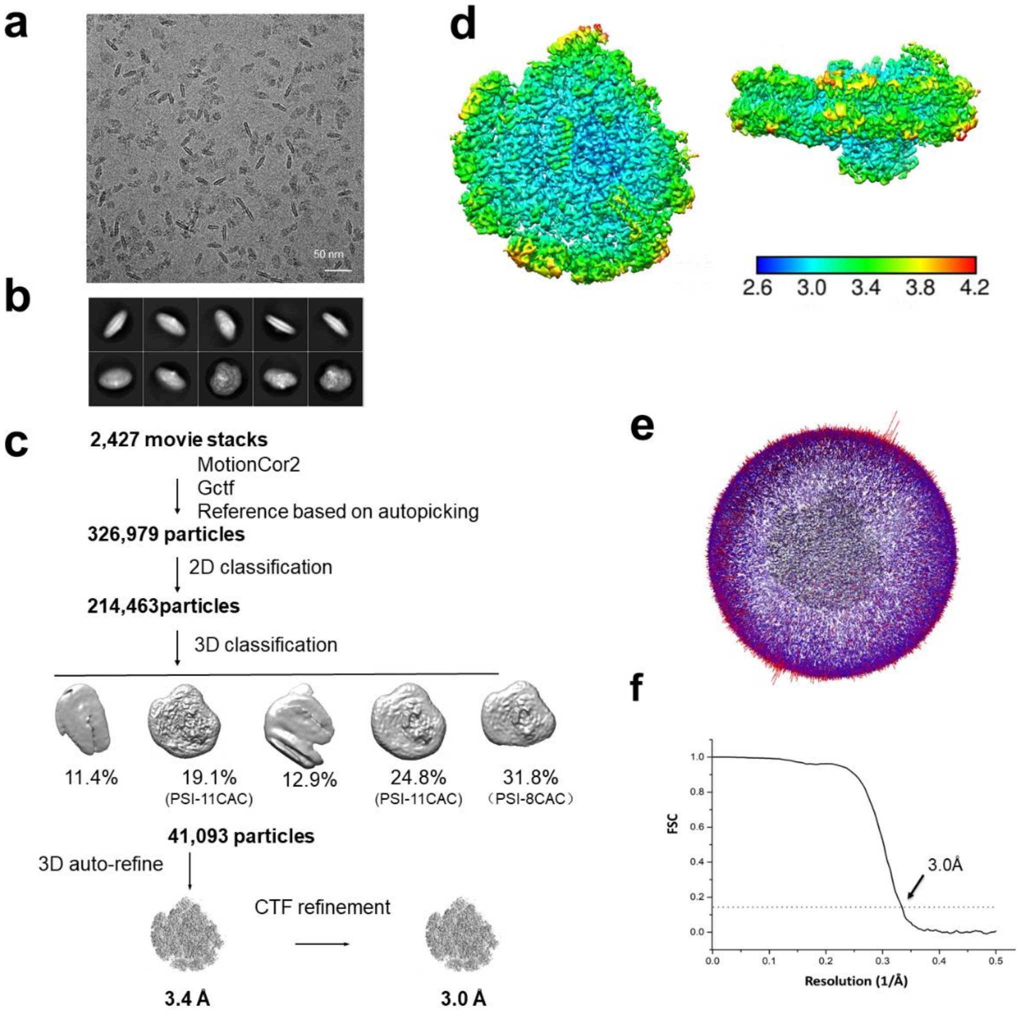

Supplementary Figure 2. Cryo-EM analysis of PSI-11CAC$_{L\text{-}phase}$ complex.

a. Representative cryo-EM micrograph of PSI-11CAC$_{L\text{-}phase}$. **b.** Representative 2D class averages. **c.** Cryo-EM data processing workflow. Different types of PSI-CAC complexes from several classes are indicated. **d.** Local resolution of the cryo-EM map estimated by ResMap. **e.** Angular distribution of the particles in the final 3D reconstruction with C1 symmetry. **f.** The Gold-standard Fourier shell correlation (FSC) curve of the density map of PSI-11CAC$_{L\text{-}phase}$ with the criterion of 0.143.

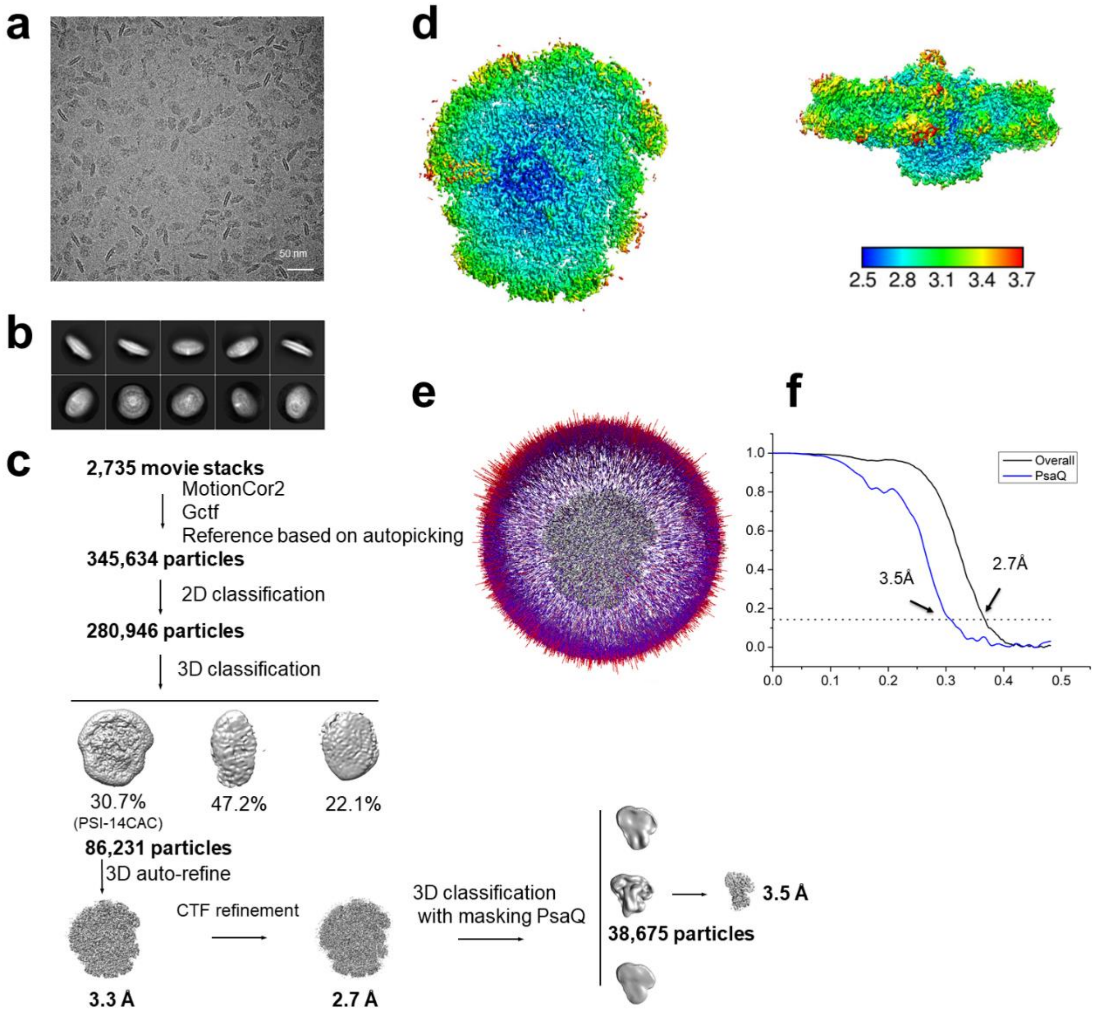

Supplementary Figure 3. Cryo-EM analysis of PSI-14CAC$_{\text{L-phase}}$ complex.

a. Representative cryo-EM micrograph of PSI-14CAC$_{\text{L-phase}}$. **b.** Representative 2D class averages. **c.** Cryo-EM data processing workflow. **d.** Local resolution of the cryo-EM map estimated by ResMap. **e.** Angular distribution of the particles in the final 3D reconstruction with C1 symmetry. **f.** The Gold-standard Fourier shell correlation (FSC) curves of the density map of PSI-14CAC$_{\text{L-phase}}$ (black line) and local map of PsaQ (blue line) with the criterion of 0.143.

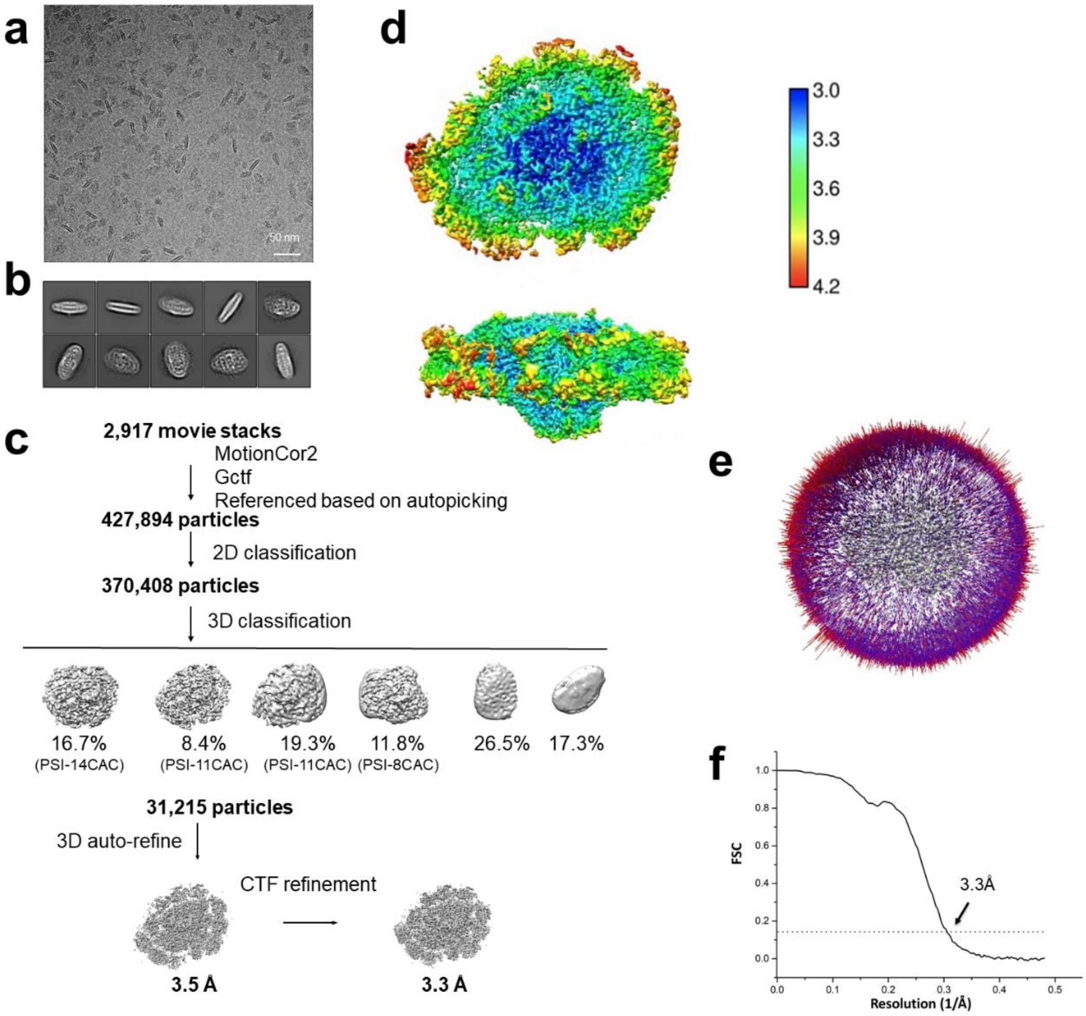

Supplementary Figure 4. Cryo-EM analysis of PSI-11CAC$_{\text{S-phase}}$ complex.

a. Representative cryo-EM micrograph of PSI-11CAC$_{\text{S-phase}}$. **b.** Representative 2D class averages. **c.** Cryo-EM data processing workflow. Different types of PSI-CAC complexes from several classes are indicated. **d.** Local resolution of the cryo-EM map estimated by ResMap. **e.** Angular distribution of the particles in the final 3D reconstruction with C1 symmetry. **f.** The Gold-standard Fourier shell correlation (FSC) curve of the density map of PSI-11CAC$_{\text{S-phase}}$ with the criterion of 0.143.

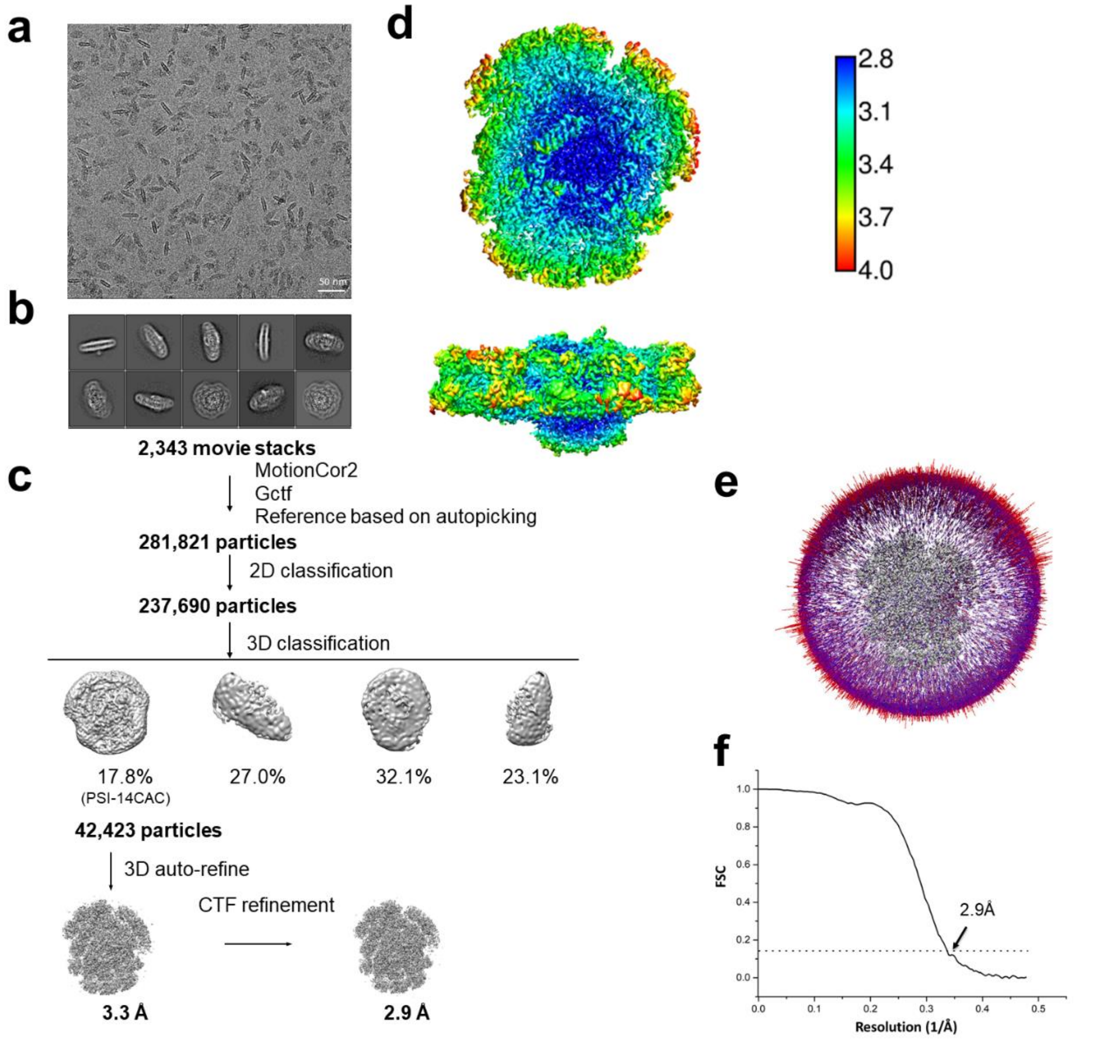

Supplementary Figure 5. Cryo-EM analysis of PSI-14CAC$_{S\text{-phase}}$ complex.

a. Representative cryo-EM micrograph of PSI-14CAC$_{S\text{-phase}}$. **b.** Representative 2D class averages. **c.** Cryo-EM data processing workflow. **d.** Local resolution of the cryo-EM map estimated by ResMap. **e.** Angular distribution of the particles in the final 3D reconstruction with C1 symmetry. **f.** The Gold-standard Fourier shell correlation (FSC) curve of the density map of PSI-14CAC$_{S\text{-phase}}$ with the criterion of 0.143.

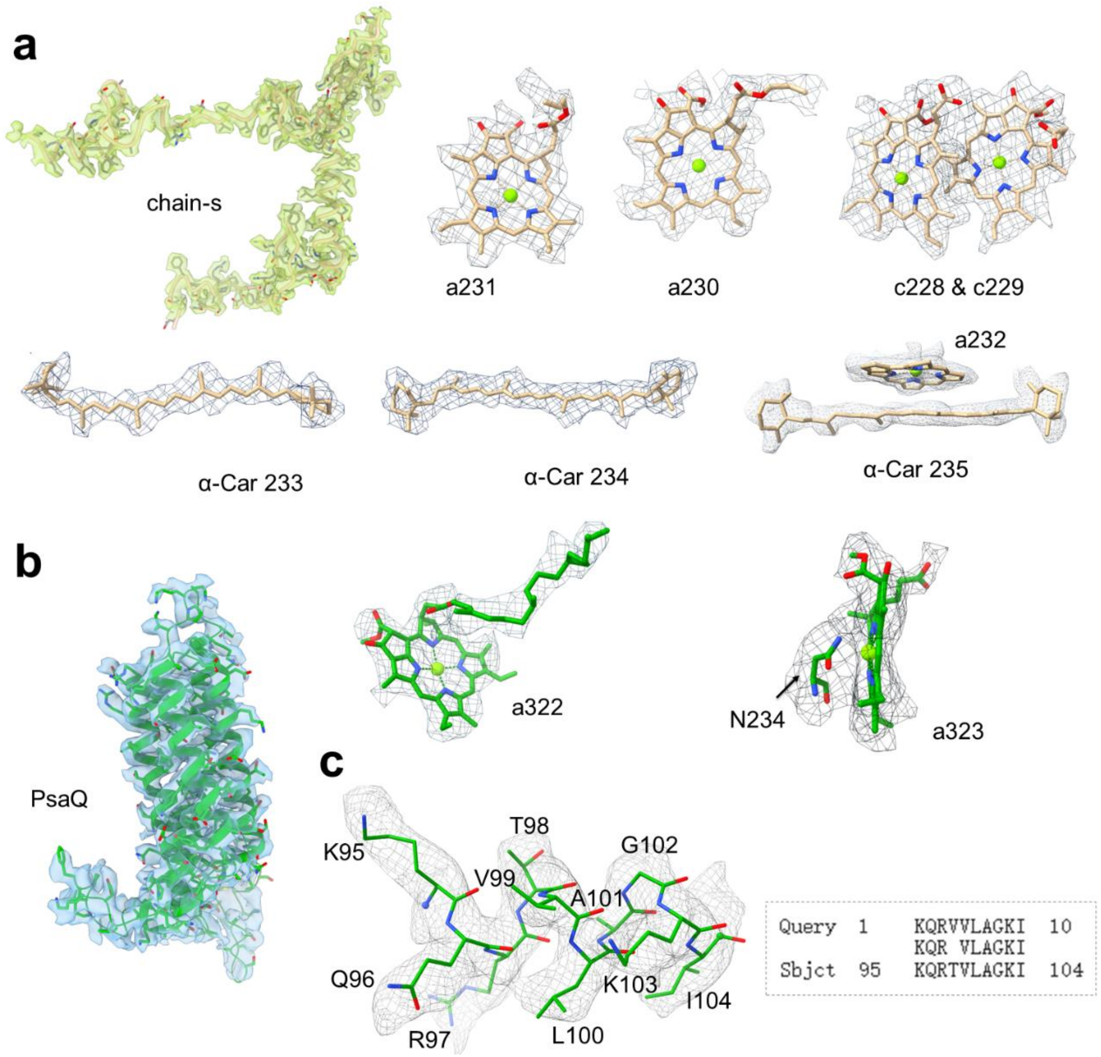

Supplementary Figure 6. Representative cryo-EM density maps of PSI-14CAC.

a. Cryo-EM densities of chain-s and its pigment molecules. **b.** Cryo-EM densities of PsaQ, two chlorophyll molecules, and N234 (indicated by a black arrow) which is the axially ligated residue of Chl a323. **c.** The identification of PsaQ. One short peptide ^{1}KQRVVLAGKI^{10} which we directly built according to the cryo-EM density shows high similarity with the fragment ^{95}KQRTVLAGKI^{104} of PsaQ.

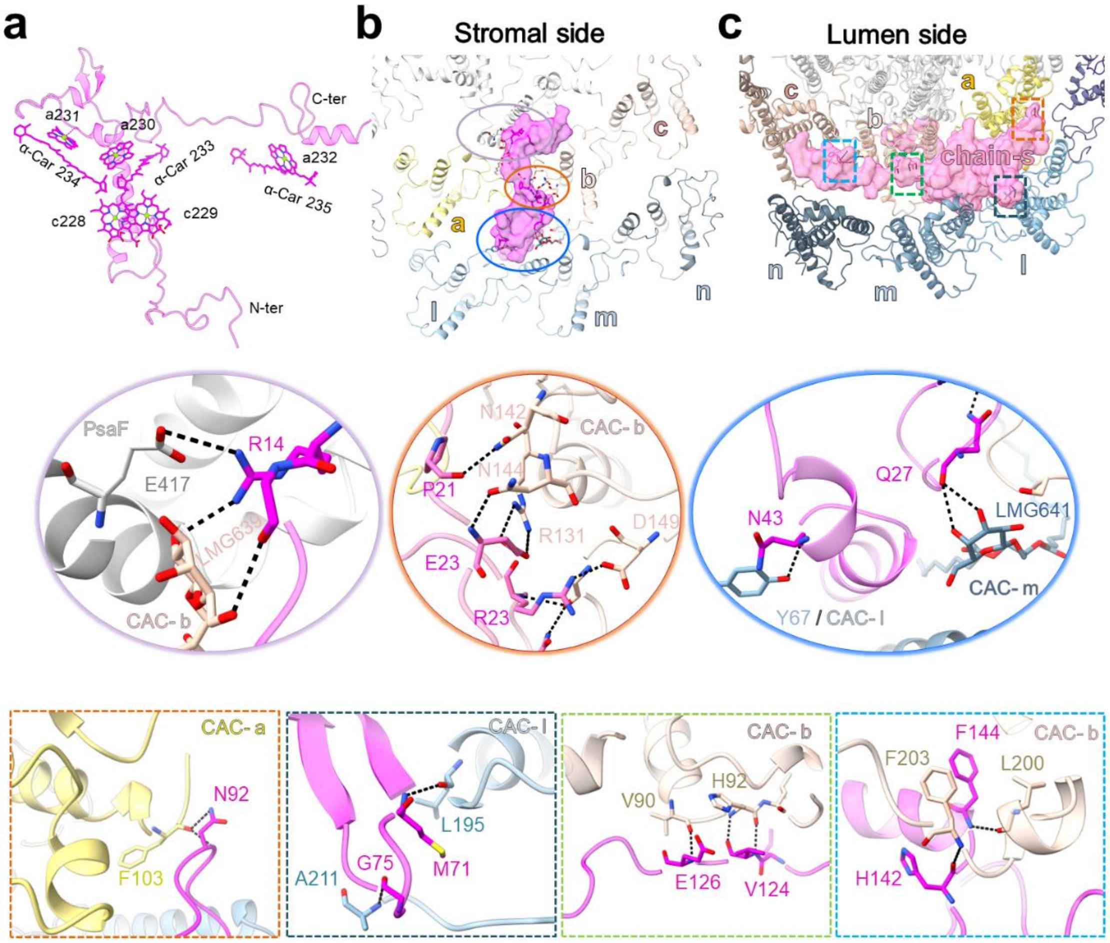

Supplementary Figure 7. Structure of chain-s and its interactions with two layers of CACs and PsaF.

a. Overall structure of chain-s. **b-c.** The chain-s forms numerous hydrogen-bond interactions with CAC-a/b/l/m and PsaF at stromal side **(b)** and lumenal side **(c)**.

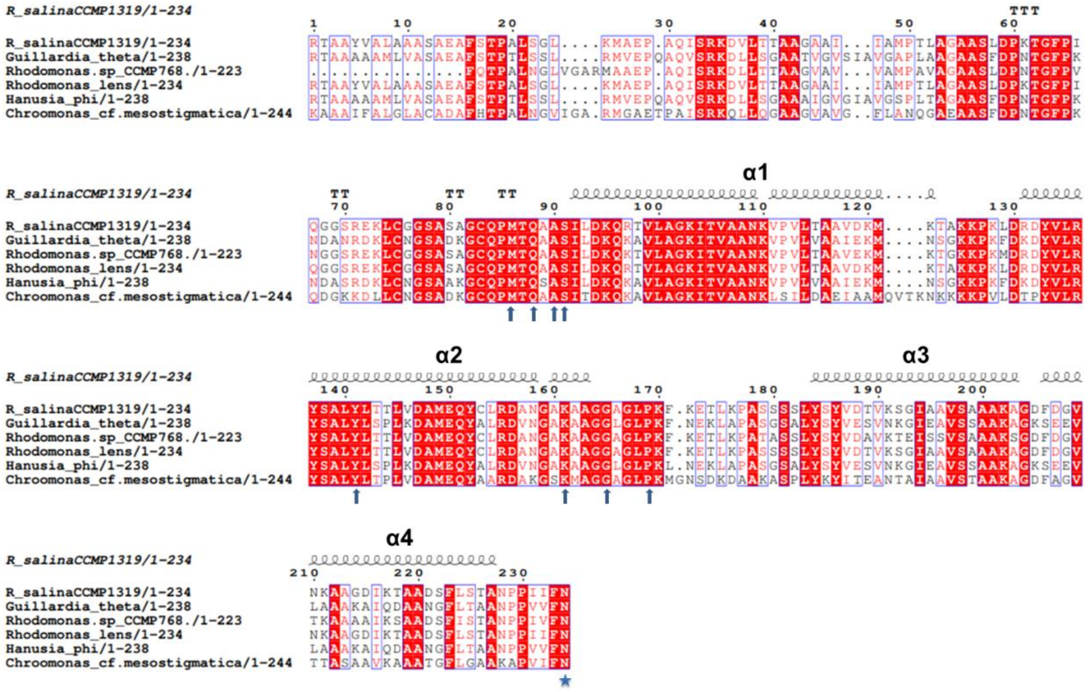

Supplementary Figure 8. Sequence alignment of *Rs*PsaQ and its homologues in cryptophytes.

Arrows indicate the conserved residues involved in the hydrogen bond interactions with PsaB and CAC-i. The conserved residue N234 which is axially ligated to chlorophyll a323 is marked with a blue star. Secondary structures are shown on the top and is referenced to *Rs*PsaQ.

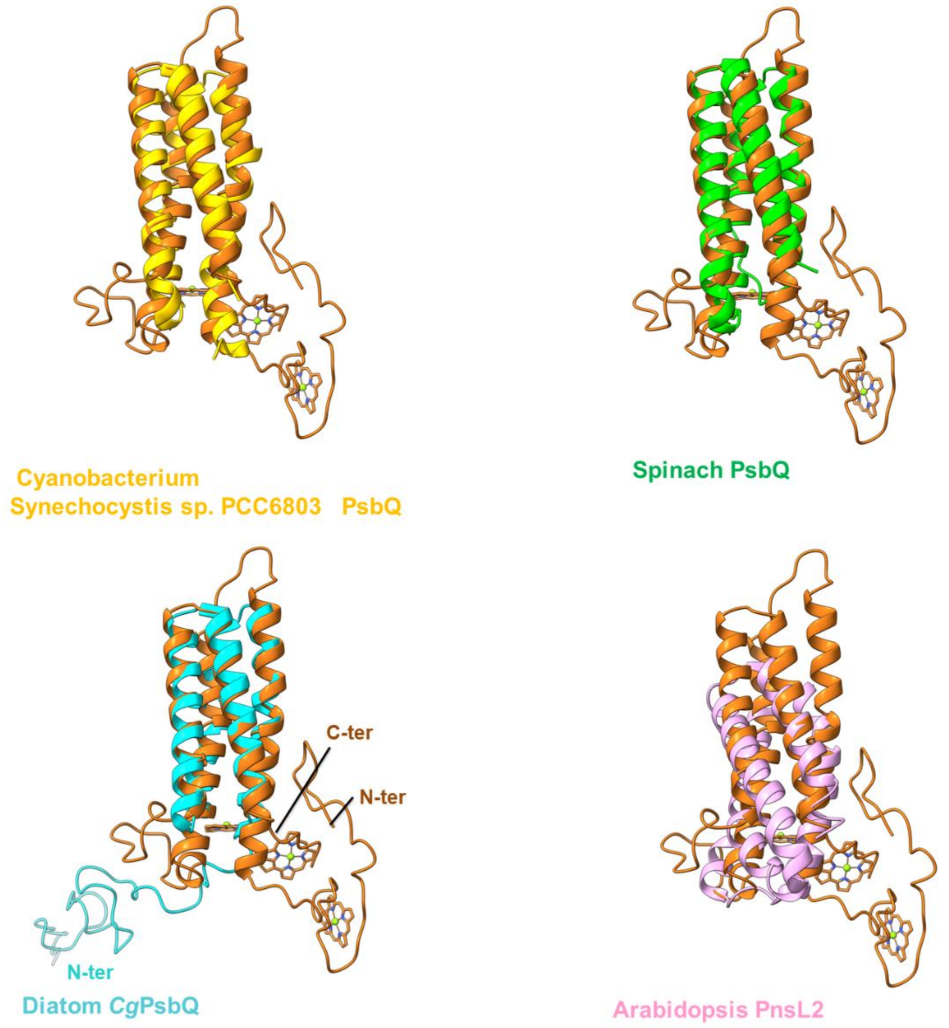

Supplementary Figure 9. Comparison of PsaQ (shown in orange) with PsbQ(-like) proteins from various phototrophs, viewed from the same direction.

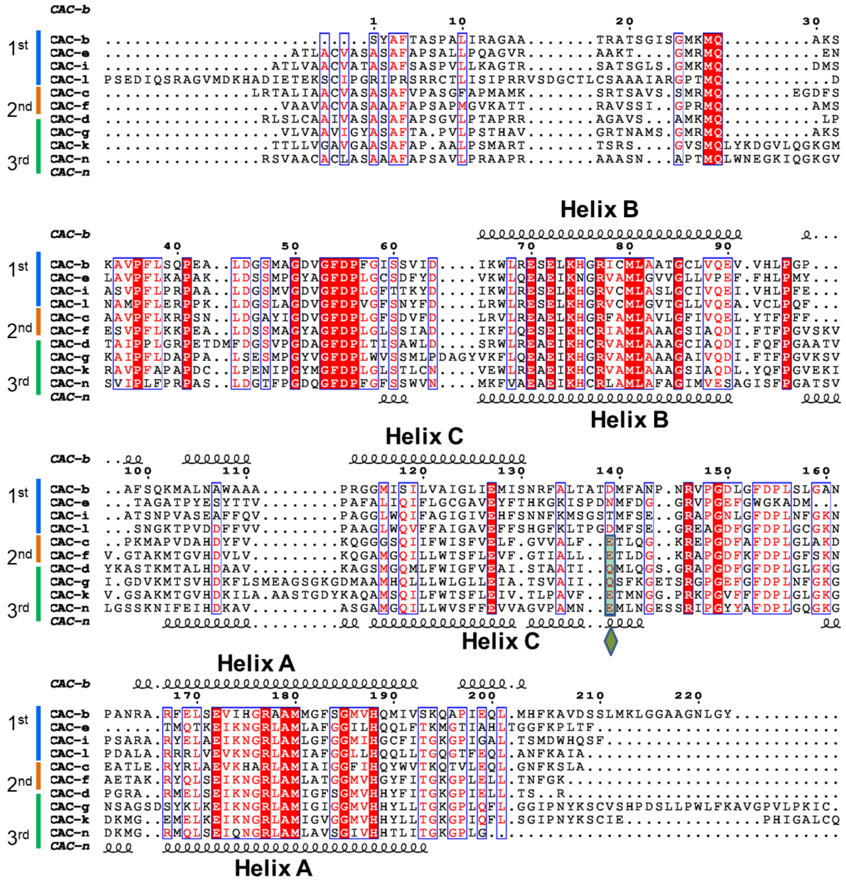

Supplementary Figure 10. Sequence alignment of CAC members from four trimers.

The sequences are divided into three groups, corresponding to the first, second and third CACs in trimers (labelled as 1st, 2nd and 3rd in the left). The top and the bottom secondary structures corresponding to CAC-b (representative of the first CACs) and CAC-n (representative of the second and third CACs) show that the first CACs have a shorter helix C than the second and third CACs. The conserved residues E/Q, which are located at the stromal end of helix C of the second and third CACs, are marked by diamond in olive green. This residue E/Q is involved in the hydrogen bond interaction with neighboring CAC within the trimer.

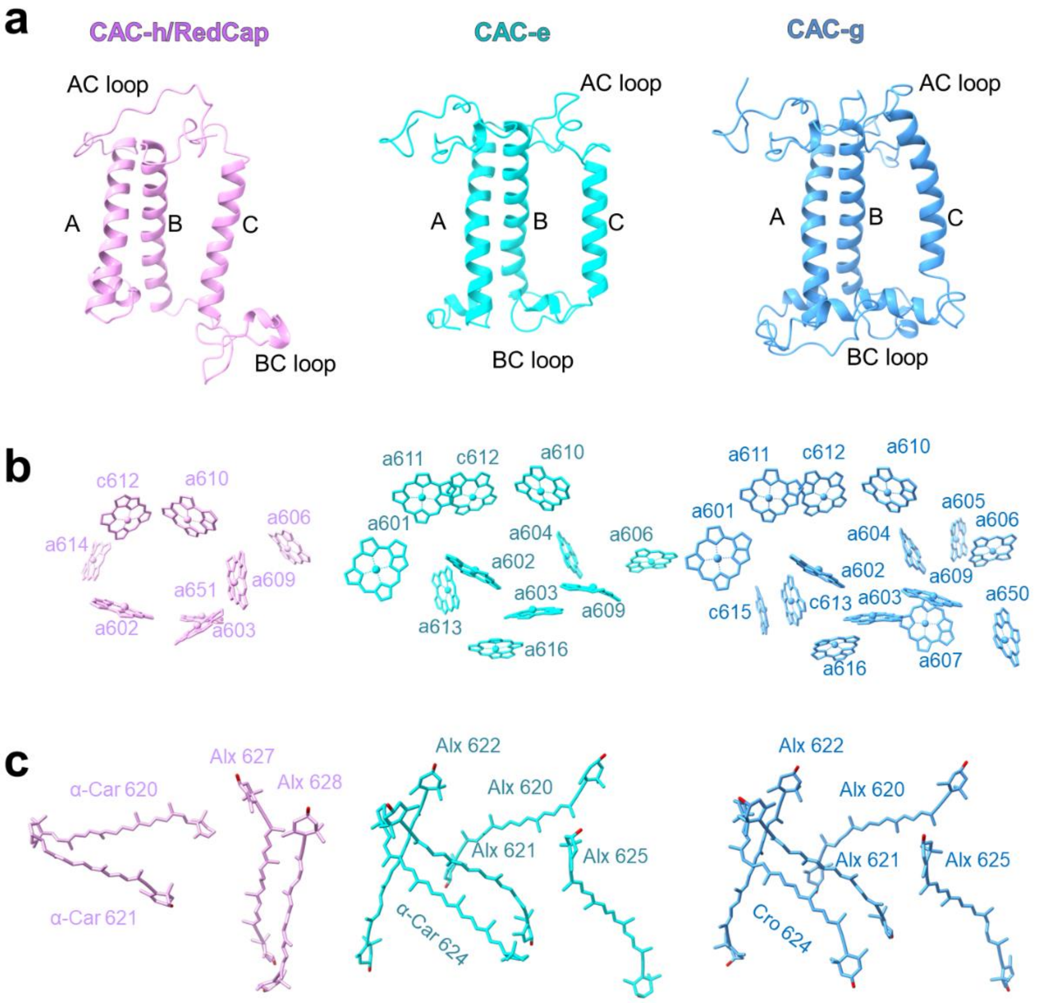

Supplementary Figure 11. Comparison of CAC-h with other CACs.

a-c. Comparison of apo-proteins (**a**), chlorophylls (**b**) and carotenoids (**c**) of CAC-h with other CACs (represented by CAC-e and CAC-g).

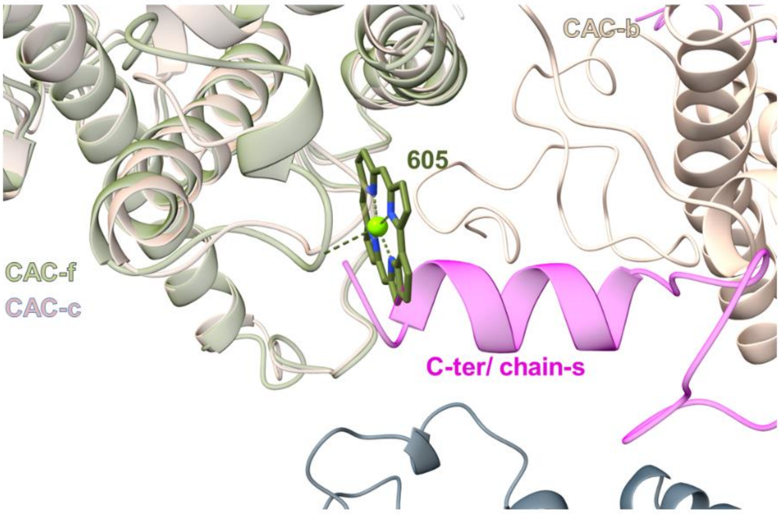

Supplementary Figure 12. Comparison of CAC-c and CAC-f in local region around Chl 605.

The Chl 605 of CAC-f clashes with the C-terminus of chain-s when superposing CAC-f on CAC-c.

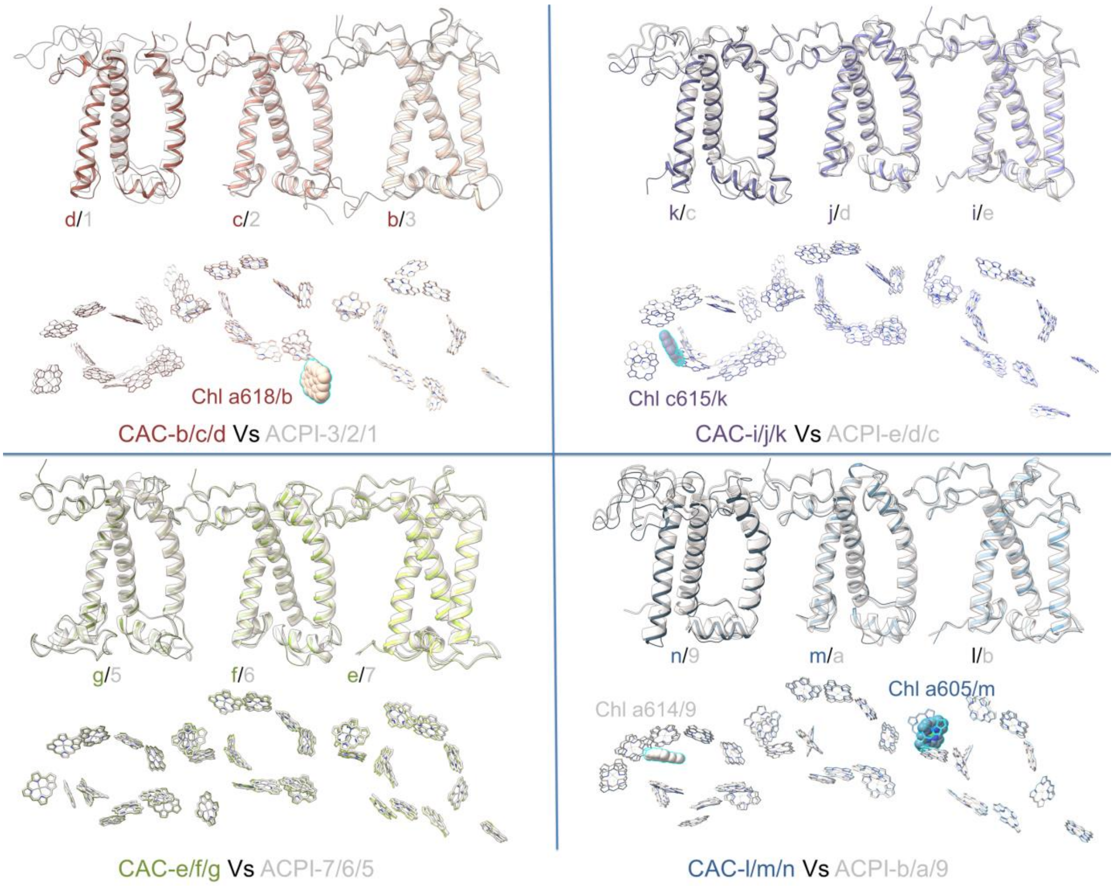

Supplementary Figure 13. Comparisons of *Rs*CAC trimers with the corresponding *Cp*ACPI trimers (PDB code 7Y7B).

The apo-protein and chlorophyll arrangement of four trimers are compared separately. CAC trimers in *Rs*PSI-CAC structure are shown in colors same to that in Fig. 1, whereas trimers in *Cp*PSI-ACPI structure are shown in light grey. The chlorophylls present in one structure but absent in another structure are shown as stick-ball mode and outlined in cyan, whereas other chlorophylls are shown as lines.

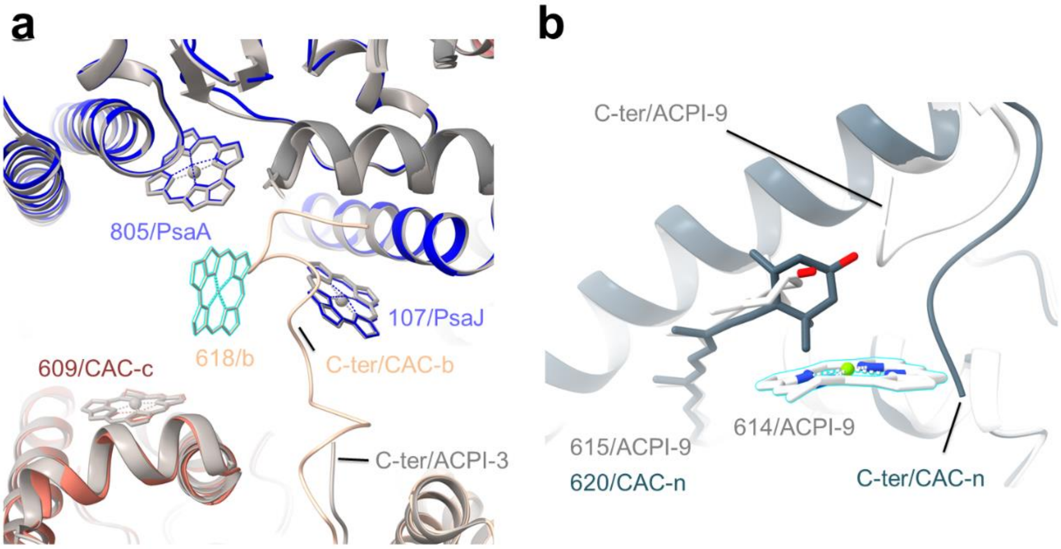

Supplementary Figure 14. Superposition of *Rs*PSI-CAC and *Cp*PSI-ACPI (PDB code 7Y7B) structures showing the locations of chllorophylls different between two complexes.

a. CAC-b in *Rs*PSI-CAC possesses a long C-terminal loop and binds Chl a618. **b.** CAC-n does not bind Chl 614 in ACPI-9 due to the steric hindrance of the C-terminal loop and Alx620 in CAC-n. Protein subunits in *Rs*PSI-CAC structure are shown in different colors, whereas *Cp*PSI-ACPI is shown in light grey. Two chlorophylls are outlined in cyan.

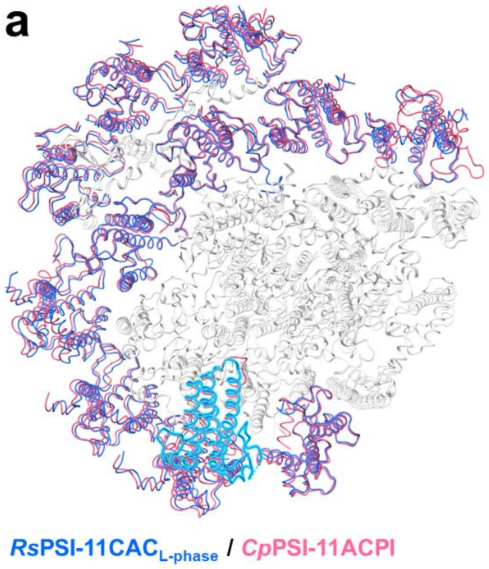

Supplementary Figure 15. Structural comparison of PSI complexes from L-phase cryptophyte cells.

a. Superposition of PSI-11CAC$_{\text{L-phase}}$ structure with *Cp*PSI-11ACPI structure (PDB code 7Y8A) viewed from the lumenal side. **b.** Superposition of PSI-14CAC$_{\text{L-phase}}$ structure with *Cp*PSI-14ACPI structure (PDB code 7Y7B) viewed from the lumenal side. The PSI core in all structures are colored grey. The antenna proteins are colored blue and pink in *Rs*PSI-CAC and *Cp*PSI-ACPI structures, respectively. PsaQ of *Rs*PSI-CAC structures is outlined in cyan.

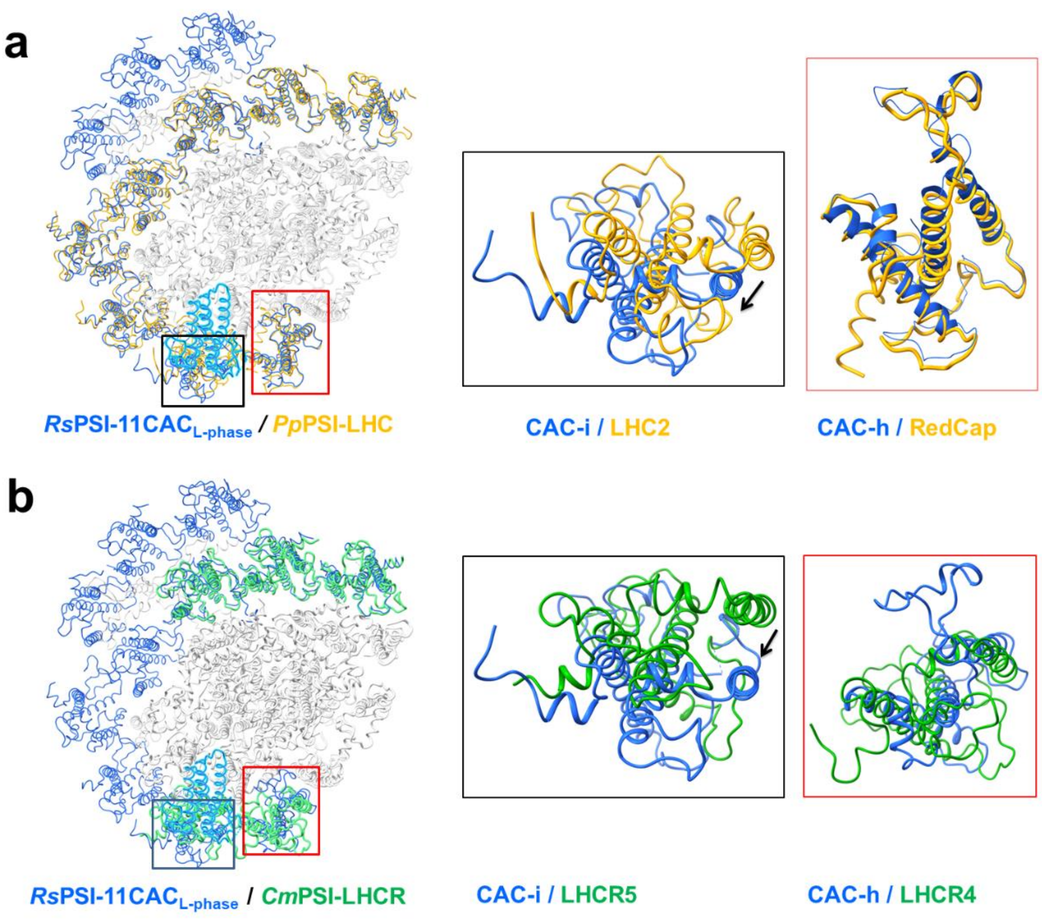

Supplementary Figure 16. Structural comparison of PSI complexes from *R. salina* and red algae.

a-b, Superposition of *Rs*PSI-11CAC$_{L\text{-phase}}$ on PSI-LHC moiety of *Pp*PBS-PSII-PSI-LHC (PDB code 7Y5E) **(a)** and *Cm*PSI-LHCR (PDB code 5ZGB) **(b)**. The PSI core in all structures are colored grey. The regions of *Rs*CAC-i and *Rs*CAC-h are indicated by black and red boxes, and shown in zoom-in view. The shift of *Rs*CAC-i comparing with the corresponding LHC antenna in *Pp*PBS-PSII-PSI-LHC and *Cm*PSI-LHCR is indicated by black arrow. The antenna proteins are colored blue, orange and green in *Rs*PSI-11CAC$_{L\text{-phase}}$, *Pp*PSI-LHC and *Cm*PSI-LHCR respectively. PsaQ of *Rs*PSI-11CAC$_{L\text{-phase}}$ is outlined in cyan.

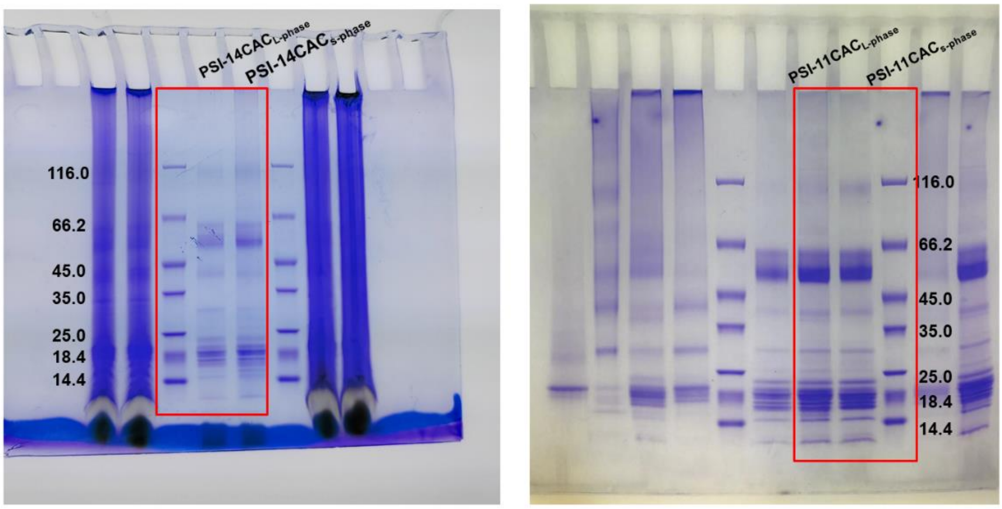

Supplementary Figure 17. Uncropped and unedited gel images for Supplementary Figure 1e.

Supplementary Table 1. Comparison of chlorophylls between *Rs*CACs and corresponding *Cp*ACPIs

CAC/ACPI	a/4	b/3	c/2	d/1	e/e	f/d	g/c	i/7	j/6	k/5	l/b	m/a	n/9	h/8
**Chlorophyll**														
**601**	a	a	a	a	a	a	a	a	a	a	a	a	a	-
**602**	a	a	a	a	a	a	a	a	a	a	a	a	a	a
**603**	a	a	a	a	a	a	a	a	a	a	a	a	a	a
**604**	a	a	a	a	a	a	a	a	a	a	a	a	a	-
**605**	-	-	-	a	-	a/-	a	-	a/c	a	-	a/-	a	-
**606**	a/c	a	a	a	a/c	a	a	a	a	a	a/c	a	a	a
**607**	-	-	a	a	-	a	a	-	a	a	-	a	a	-
**609**	a	a	a	a	a	a	a	a	a	a	a	a	a	a
**610**	a	a	a	a	a	a	a	a	a	a	a	a	a	a
**611**	a	a	a	a	a	a	a	a	a	a	a	a	a	-
**612**	a	a	c	c	c	c	c	c	c	c	c	c	c	a
**613**	a	a	a	a	a	a	c/a	a	a	c/a	a	a	a	-
**614**	-	-	-	-	-	-	-	-	-	-	-	-	-	a
**615**	-	-	-	-	-	-	c	-	-	c/-	-	-	-	-
**616**	a	a	a	a	a	a	a	a	a	a	a	a	a	-
**617**	-	a/c	-	-	-	-	-	-	-	-	-	-	-	-
**618**	-	a/-	-	-	-	-	-	-	-	-	-	-	-	-
**650**	-	-	-	-	-	-	a/-	-	-	-	-	-	-	-
**651**	-	-	-	-	-	-	-	-	-	-	-	-	-	a
**652**	-	-	-	-	-	-	-	c	-	-	-	-	-	-
	-	-	-	-	-	-	-	-	-	-	-	-	-/614a	

Different chlorophylls between *Rs*CACs and *Cp*ACPIs are highlighted in red.

Supplementary Table 2. Summary of the designation of peripheral antennae and the two newly identified proteins in *Rs*PSI and *Cp*PSI structures.

R.salina		***C. placoidea***	
Name	PDB-chain ID	Name	PDB-chain ID
CAC-a	a	ACPI-4	4
CAC-b	b	ACPI-3	3
CAC-c	c	ACPI-2	2
CAC-d	d	ACPI-1	1
CAC-e	e	ACPI-11	e
CAC-f	f	ACPI-10	d
CAC-g	g	ACPI-9	c
CAC-h	h	ACPI-8	8
CAC-i	i	ACPI-7	7
CAC-j	j	ACPI-6	6
CAC-k	k	ACPI-5	5
CAC-l	l	ACPI-14	b
CAC-m	m	ACPI-13	a
CAC-n	n	ACPI-12	9
chain-s	s	ACPI-S	Z
PsaQ	Q	Unk1	X
